# 528. Matched Retrospective Study, Comparing Molnupiravir to Nirmatrelvir-Ritonavir (Paxlovid) in the Treatment of Mild-Moderate COVID-19 in Immunocompromised Cancer Patients

**DOI:** 10.1093/ofid/ofad500.597

**Published:** 2023-11-27

**Authors:** Andrea Haddad, Mohammad Moussa, Ray Y Hachem, Ying Jiang, Hiba Dagher, Patrick Chaftari, Anne-Marie Chaftari, Issam I Raad

**Affiliations:** UT MD Anderson Cancer Center, houston, Texas; MD Anderson Cancer Center, Houston, Texas; MD Anderson UT, Houston, Texas; UT MD Anderson Cancer Center, houston, Texas; UT MD Anderson Cancer Center, houston, Texas; UT MDAnderson Cancer Center, Houston, TX; MD Anderson UT, Houston, Texas; MD Anderson UT, Houston, Texas

## Abstract

**Background:**

Paxlovid has been shown to reduce the risk of progression (hospitalization/death) by 88% as compared to placebo, while molnupiravir reduced that risk by 31%. However, these two agents have not been compared in the same study patient population. We therefore conducted a matched controlled study, comparing molnupiravir to paxlovid in terms of safety (adverse events and drug-drug interactions) and efficacy (progression and rebound events) in the treatment of mild-moderate COVID-19 in immunocompromised cancer patients.

**Methods:**

This is a single center, retrospective study that included 240 cancer patients diagnosed with COVID-19 in 2022. Patients who received molnupiravir were matched to those who received paxlovid on a 1:2 ratio, based on age group (18-64 years vs. ≥ 65) and type of cancer (lung cancer, hematologic malignancy, or other solid tumors). We collected patient demographics, comorbidities, malignancy history and treatment, vaccination status, as well as post-treatment progression and safety information. We used margins of 10% with 95% confidence intervals to determine the equivalence of outcomes.

**Results:**

As shown in table 1, the two groups had comparable demographics, underlying malignancies, and most comorbidities, however smoking history, underlying CKD, and CAD were more common in the molnupiravir group. Similarly presenting symptoms were comparable, but cough, dyspnea and sore throat were more prevalent in the molnupiravir group.

By multivariate analysis, 3 factors were independently associated with progression: hypertension, cough on presentation and failure to complete the 5-day course of antiviral therapy - P < 0.037. However, multivariate analysis failed to show any difference between the 2 groups in terms of progression to severe/disease and rebound events with equivalent margins within 10%.

Patients who received paxlovid however were significantly more prone to drug-drug interactions/adverse events (28% vs. 0 - P < 0.0001).

**Figure:**
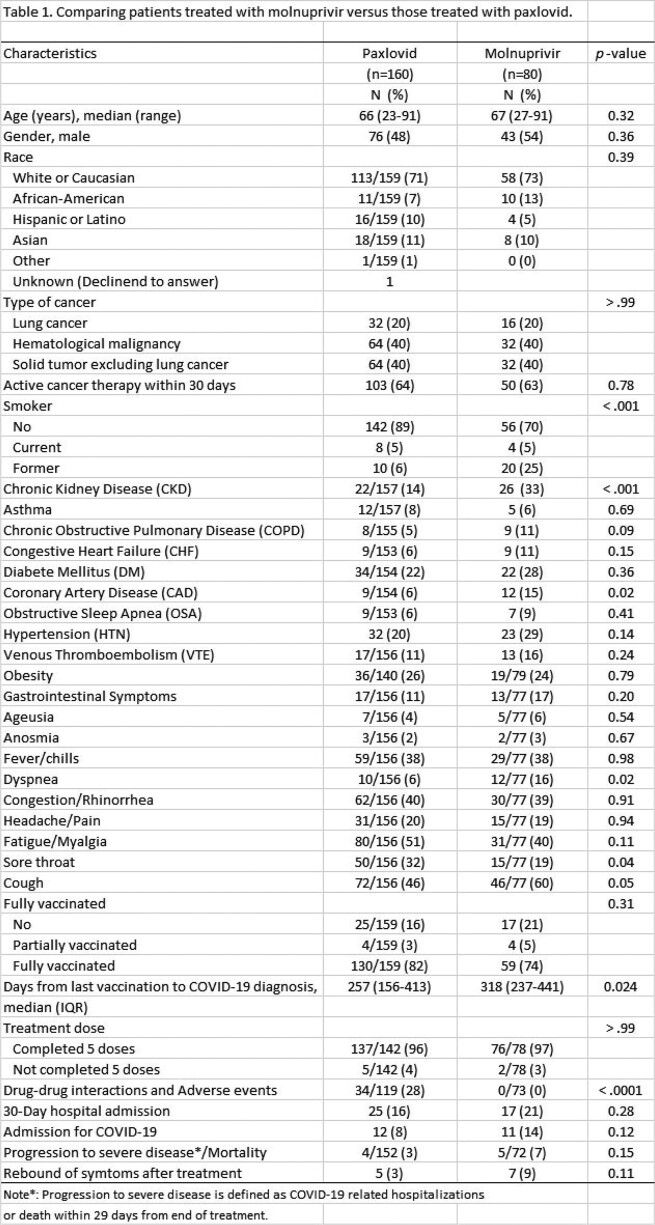

Comparing patients treated with molnuprivir versus those treated with paxlovid

**Conclusion:**

In the treatment of mild to moderate COVID-19 in cancer patients, molnupiravir was comparable to paxlovid in preventing progression to severe disease/death and rebound events, with a superior safety profile.

**Disclosures:**

**Issam I. Raad, Distinguished Professor**, Novel Anti-Infective Technologies, LLC: Technology License

